# Validity and Reliability Analysis of Knowledge of, Attitude toward and Practice of a Case-mix Questionnaire among Turkish Healthcare Providers

**DOI:** 10.36469/9891

**Published:** 2014-08-06

**Authors:** Saad Ahmed Ali Jadoo, Seher Nur Sulku, Syed M. Aljunid, Ilker Dastan

**Affiliations:** 1 United Nation University–International Institute of Global Health (UNU-IIGH), Kuala Lumpur, Malaysia; International Center for Casemix and Clinical Coding (ITCC), Kuala Lumpur, Malaysia; 2 Department of Econometrics, Economics and Management Sciences Faculty Gazi University, Ankara, Turkey; 3 International Center for Casemix and Clinical Coding (ITCC), Kuala Lumpur, Malaysia; 4 Department of Economics Izmir University of Economics, Izmir, Turkey

**Keywords:** validity, reliability, practice, knowledge, diagnosis related group, case-mix, attitude

## Abstract

**Objectives:** This study was aimed to assess validation and reliability of knowledge of, attitude toward and practice (KAP) of a Case-mix and Diagnosis Related Group (DRG) system questionnaire.

**Methods:** A sample of 238 health care providers selected conveniently from three public hospitals in Turkey was enrolled in a cross-sectional study from September 1 until November 30, 2012. The mean age was 38.63 years (standard deviation [SD] 10.52), ranging from age 21 to 60 years. More than one-half were males (52.1%), nearly two-fifths were medical doctors (39.9%), one-third were nurses (33.2%), one-sixth were auxiliary staff (16.4%) and the remaining were coders (10.5%). Only one-third (33.6%) of respondents attended a workshop or training program in the Case-mix or DRG system. After examining content validity, factor analysis was conducted, internal consistency of the questionnaire was assessed by Cronbach’s alpha estimate, and test-retest reliability was evaluated.

**Results:** The sample adequacy for extraction of the factors was confirmed by the Kaiser-Meyer-Olkin test (0.915) and the Bartlett test (1052). Factor analysis showed three factors, including attitude (36.43%), practice (23.39%) and knowledge (17%), with a total variance of 76.82%. The reliability of each section of the questionnaire was as follows: knowledge (0.963), attitude (0.964) and practice (0.973). Cronbach’s alpha total was 0.941, which showed excellent internal consistency.

**Conclusions:** This study demonstrated that the designed questionnaire provided high construct validity and reliability, and could be adequately used to measure KAP among health care staff of the Case-mix and DRG system in Turkey.

## INTRODUCTION

Case-mix is a classification system that classifies hospital cases into specific groups expected to have similar hospital resource use.[1] In the beginning it was created by researchers at Yale University in the late 1960s to help monitor quality of care and service use in hospitals in the United States.[2] The Diagnosis Related Group (DRG) is the most common case-mix system. The DRG system was designed to classify inpatients treated in acute hospitals to improve hospital management.[3] The DRG system has been modified and used by different countries. The DRG classification system describes the normal or typical case in a DRG i.e. allows us to define and measure the hospital's case-mix.[4] The marked increase in health care expenditure since mid of 1970s led to the emergence an alternative healthcare funding mechanism. The case-mix system and DRG classification system were promising tools, which later became the most popular provider payment mechanism for inpatient care services.[5]

Since 2003, Turkey has implemented health sector reforms, namely the health transition program (HTP). These reforms aimed to meet the Turkish citizen's priorities and expectation regarding the healthcare system, by improving the health services delivery system via strengthened financial and organizational structure of the health sector.[6,7] A Pay for performance system was initiated in 2004 to increase the productivity of health personnel by providing wage incentives.[8] In 2008, the Social Security Institution (SSI) became a single organization on the purchasing side of health care services, to eliminate fragmentation in the health financing system. A universal health insurance system has been introduced to enhance the equity and access to health care services for all citizens.[9]

Throughout this reform process, total health expenditure has increased significantly and its share on GDP reached to 6.1% in 2011 from 5.4% in 2002.[10] Recently the Turkish SSI aimed to adopt the Australian DRG payment system, to coordinate health payments and reduce hospital budgets through an adequate and efficient resource distribution system. In Turkey, studies on the DRG system were initiated in 2005 as a joint project of Hacettepe hospital, the Ministry of Health (MoH) and other associated ministries. By March 2012, 555 public hospitals of the MoH were paid partially based on DRG. When the DRG system has been completely adapted to the Turkish health sector, all SSI reimbursements to all health providers will be done through the system.[11]

Application of the DRG system requires an extensive health and treatment output classification system, and should be updated thoroughly up to any changes in medical practices. Key elements of DRG implementation at hospitals are the physicians who form the medical data in inpatient files and clinical coders who convert these data into codes. Thus, this complicated DRG system can only be employed with an efficient, organized automated system and well-informed health care staff.[12] Lack of knowledge and misunderstanding of the DRG system among health care staff and coders in hospitals would lead the system to failure. Therefore, it is important to assess the KAP levels of Turkish health care providers who will implement the DRG system. Consequently, in this study we aimed to construct a reliable questionnaire to assess the KAP of health care staff who use the DRG system in Turkey.

In fact, with the growing number of countries implementing the case-mix and DRG system, the need to evaluate these systems became inevitable. Most studies have focused on issues such as the general evaluation of Case-mix using the DRG system,[13,14] patient-level data utilization,[15] information technology (IT),[16] investigation of physicians' attitudes concerning the implementation of international classification systems of diseases,[17] improving the coding process,[18] costing methodologies,[19] clinical pathways,[20] developing of cost weights and the related base rates,[21] etc. However, little has been done to test the health care provider knowledge, attitude and practice for the Case-mix system worldwide.[22] In the case of Turkey, it was planned to use the DRG system as the main financing system in the Turkish health sector. This study was designed to assess the validation and reliability of the Case-mix and DRG system using the KAP questionnaire.

## MATERIALS AND METHODS

### Item Development

This study was cross-sectional and designed to validate the KAP Case-mix questionnaire in Turkey, from September 1 to November 30, 2012. The study tool was developed by an expert team from the International Center for Case-mix and Clinical Coding (ITCC) in the Medical Center of the National University of Malaysia (UKMMC). The questionnaire was prepared to assess the validation and reliability of KAP on the Case-mix and DRG system. Four sections were included in this questionnaire:

1) **Demographic Factors:** Intended to discover the sociodemographic characteristics such as age, gender and occupation in addition to one question (yes/no) regarding the respondent's attendance at a workshop or training program for the Case-mix and DRG system.
2) **Knowledge:** Defined as respondent's knowledge of the Case-mix system in general, DRG system, Case-mix grouper, Case-mix tariff, cost weight and so on. This domain consists of 10 questions.
3) **Attitude:** Defined as respondent's opinions about issues such as the need for hospital costing data, IT infrastructure and trained staff; the role of Case-mix, for example, in improving hospital efficiency and its effect on clinical staff workload and their imbursement. This domain contains 12 items.
4) **Practice:** Defined as respondent's practice of Case-mix implementation, such as the coding process and the importance of patient administration and clinical data, costing data and costing strategy, clinical pathway and so on. This domain consists of 10 items.

The KAP questionnaire responses were given on a five-point Likert type scale ranging from (1) "strongly agree" to (5) "strongly disagree." Negatively worded questions were reverse scored (so that 1 = 5, 2 = 4, etc.).

### Content Validity

The questionnaire was translated to Turkish language, and the content validity was assessed by a committee of four Turkish experts in the field with the required competence in English language and familiarity with the topic: public health specialist, IT specialist, health economist and Case-mix specialist. The questionnaire was pre-piloted with a randomly selected sample of 20 health care providers at the Ankara Numune Training and Research Hospital (Ankara City) for appropriateness of language and sensitivity of questions.

### Empirical Study

The content validated in the Turkish version was distributed among 403 respondents. The sample was calculated assuming that the knowledge is 50% among health care providers in Turkey using the formula:

$$N = [Z\alpha^2 \times P \times Q \; / \; (M.E.)^2].$$

This was also tested by a pilot test. So, $n = (1.96)^2 \times (0.50) \times (0.50)/(0.05)^2 = 384$. Non-response correction = 5%. Thus, the total sample size with provision for drop-outs from the study = 384 + 5% of 403(19.0) = 403. The total number of health care providers who answered the self-administered questionnaire was 238, making the response rate of the study 59%. Three public hospitals that have recently begun to implement the DRG system were selected conveniently. The first two hospitals have great experience in research and training, providing a wide range of medical and surgical specialties in a capacity of 1,140 beds and 900 beds, respectively. The third was a state hospital providing a primary emergency reception centre for the rural population in over 200 inpatient beds, supported by extensive outpatient and community-based services.Trained interviewers and data collectors were recruited to explain the objectives and conditions of the study to respondents. A convenience sampling technique was used to select the study sample. Each eligible respondent received one translated version of the questionnaire, which was collected back 1 week later by data collectors. All health care providers who were employed in the selected hospitals during the study period and willing to participate were included. Absent and transient contract employees were excluded.

### Construct Validity

Data collected from an empirical study was used to test the construct validity of the questionnaire. Exploratory factor analysis using the varimax rotation method (principle component analysis [PCA])[23,24] was used to determine the structure of the questionnaire, namely the KAP sub-scales measured by this questionnaire. The criterion validity of the questionnaire was not checked, as a standardization tool for assessment of health care workers' KAP of Case-mix or DRG systems in Turkey has not yet been proposed.

### Internal Consistency and Reliability

Finally, the internal consistency of the questionnaire and of each sub-scale derived from the construct validity was tested using Cronbach's alpha coefficient. This coefficient ranges from 0 to 1. Large Cronbach's alpha values indicate a high consistency of the questions, of which the sub-scale consists.[25] The questionnaire's reliability was also assessed by calculating Intra-class Correlation Coefficient (ICC), which takes values between -1 and +1. The values proximate (+1) to show high repeatability of the questionnaire. The results are shown as ICC (95% confidence interval [CI]).[26,27] A convenient sample of 60 health care providers completed the questionnaire twice, with an interval of 2 weeks, in order to examine the stability of the scale.[28]

### Ethical Approval

Our study protocol was approved by the Ministry of Health, Turkey, code number (B.10.0 SHG.0.20.00.00.-01099/19920), 16 August 2012, and by respective authorities of the selected hospitals where data collection took place. A brief explanation of the study's objectives was given to all respondents. Confidentiality was assured and written consent was obtained from all respondents.

### Statistical Analysis

Evaluation for normality of distribution of the continuous variables was tested by the Kolmogorov-Smirnov test and all the quantitative data were found to be normally distributed. The categorical data are presented as absolute and relative frequencies (%), while the continuous data are presented as mean values ± standard deviations (SDs). The suitability of the data for carrying out such analysis was tested by using the Bartlett sphericity test and the Kaiser-Meyer-Olkin (KMO) statistic test that evaluates the degree of correlation among the questions included in the questionnaire. The exploratory factor analysis using PCA followed by varimax rotation was used to evaluate the construct validity of the KAP questionnaire. The criterion of Kaiser (eigenvalue ≥1) and screen plot were used to determine the number of factors to be extracted. An orthogonal rotation (i.e. varimax) was used to improve the explanatory ability of the factors. Each factor that emerged was interpreted based on questions with load values >0.4. All statistical analysis was carried out using the SPSS program, version 16 (SPSS Inc, Chicago, IL, USA).[29]

## RESULTS

### Descriptive Results

Table 1 presents the baseline characteristics of the participants. A total of 238 health care providers were included in the study. The mean age was 38.63 years (SD: 10.52), ranging from age 21 to 60 years. The highest response rates (33.6%) were among those age 31-40 years. More than half were males (52.1%), nearly two-fifth were medical doctors (39.9%), one third were nurses (33.2%), one-sixth were auxiliary staff (16.4%) and the remaining were coders (10.5%). It was observed that only one-third (33.6%) of respondents attended a workshop or training program regarding Case-mix or DRG systems. Appendix 2 illustrates the response rate according to occupation and the frequencies of attendance at an education program in each occupation group.

**Table 1.** Frequency Distribution of Socio-demographic Characteristics of Respondents (n=238)

| No. | Respondents' Characteristics | | Frequency | % |
|---|---|---|---|---|
| 1. | **Age group, years** | 21-30 | 60 | 25.2 |
| | | 31-40 | 80 | 33.6 |
| | | 41-50 | 56 | 23.5 |
| | | 51+ | 42 | 17.6 |
| 2. | **Gender** | Male | 124 | 52.1 |
| | | Female | 114 | 47.9 |
| 3. | **Occupation** | Medical doctor | 95 | 39.9 |
| | | Nurse | 79 | 33.2 |
| | | Auxiliaries | 39 | 16.4 |
| | | Coders | 25 | 10.5 |
| 4. | **Training program** | Attended | 80 | 33.6 |
| | | Not attended | 158 | 66.4 |

### Content Validity

The four experts reported that the statements were clear, understandable and in a logical order. The twenty participants in the first pilot study (average age: 44.60 years, SD: 10.05; age range: 27-58 years) agreed on all items and the related domains. According to the findings in the pilot study, the committee suggested keeping the same items of both original and translated forms with some changes in the translation to make it clearer and understandable.

### Construct Validity

The sample adequacy for extraction of the factors was confirmed. The KMO test (0.915) and the Bartlett test of sphericity ($\chi^2$= 496; p<0.001) showed that the data met the criteria required for factor analysis.[30] Three potential factors that explained 76.82% of the variance were identified. All factor loadings were >0.4, indicating that they were statistically significant and higher than the recommended level. The factor loading of each item is listed in Table 2. The first factor included 12 items related to attitude toward the Case-mix system that explained 36.43% of the total variance. The second factor included 10 items related to Case-mix practices that explained 23.39% of the total variance. The last factor comprises 10 items related to knowledge of the Case-mix system that explained 17.0% of the total variance.

**Table 2.** Rotated Factors Loading

| Items Code | Factor 1 | Items Code | Factor 2 | Items Code | Factor 3 |
|---|---|---|---|---|---|
| Attitude 1: | 0.934 | Practice 1: | 0.974 | Knowledge 1: | 0.925 |
| Attitude 2: | 0.928 | Practice 2: | 0.960 | Knowledge 2: | 0.921 |
| Attitude 3: | 0.920 | Practice 3: | 0.945 | Knowledge 3: | 0.918 |
| Attitude 4: | 0.884 | Practice 4: | 0.927 | Knowledge 4: | 0.918 |
| Attitude 5: | 0.872 | Practice 5: | 0.919 | Knowledge 5: | 0.917 |
| Attitude 6: | 0.857 | Practice 6: | 0.914 | Knowledge 6: | 0.913 |
| Attitude 7: | 0.850 | Practice 7: | 0.887 | Knowledge 7: | 0.908 |
| Attitude 8: | 0.804 | Practice 8: | 0.869 | Knowledge 8: | 0.773 |
| Attitude 9: | 0.772 | Practice 9: | 0.817 | Knowledge 9: | 0.655 |
| Attitude 10: | 0.770 | Practice 10: | 0.670 | Knowledge 10: | 0.639 |
| Attitude 11: | 0.698 | | | | |
| Attitude 12: | 0.695 | | | | |

Only items with factor loading >0.30 are shown. Factor 1 indicates Practice; factor 2, Attitude; factor 3, Knowledge. The three factors that are probably present among the items together explain 76.82% of the total variance in the results.

### Reliability - Internal Consistency

Cronbach's alpha coefficients were excellent for knowledge of (0.963), attitude toward (0.964) and practice of (0.973) Case-mix and DRG systems. As a whole, the questionnaire's internal consistency (Cronbach's α coefficient 0.941) and the ICC (0.930) were also excellent, indicating an appropriate stability of the questionnaire and sufficient reliability (Table 3).

**Table 3. Cronbach's α Coefficient and ICC for the entire KAP Scale and its Subscales (n = 238)**

| Subscales | No. of Items | Mean (SD) | Cronbach's α Coefficient | ICC (n = 60) | 95% CI |
|---|---|---|---|---|---|
| **Knowledge** | 10 | 25.93 (12.22) | 0.963 | 0.956 | 0.956-0.970 |
| **Attitude** | 12 | 28.80 (13.66) | 0.964 | 0.956 | 0.956-0.970 |
| **Practice** | 10 | 24.63 (12.37) | 0.973 | 0.967 | 0.967-0.978 |
| **Total scale (n=238)** | 32 | 79.37 (26.23) | 0.941 | 0.930 | 0.930-0.951 |

SD: standard deviation; ICC: Intra-class Correlation Coefficient; CI: confidence interval

### Reliability – Test-Retest

A paired samples t-test was run on a sample of 60 health care providers (mean age 37.42 years, SD: 9.86; age range 24-57 years) to determine whether there was a statistically significant mean difference and statistically significant correlation between KAP and the DRG system when participants responded twice within an interval of 2 weeks. According to the results in Table 4, there is a significant positive correlation between two measurements ($r = 0.972$, $p<0.001$) and there is no difference between the means of two measurements ($p>0.05$).

**Table 4.** Test-retest Reliability between Two Measurements (n = 60)

| Test Mean | (SD) | Re-test Mean | (SD) | n | r | P* | df | t | P** |
|---|---|---|---|---|---|---|---|---|---|
| 81.483 | 24.312 | 81.833 | 20.317 | 60 | 0.972 | 0.000 | 59 | - 0.410 | 0.683 |

*P<0.001, **p>0.05; SD: standard deviation

## DISCUSSION

The present study is an attempt to assess validity and reliability of a KAP questionnaire in Case-mix and DRG systems among health care providers in Turkey. The KAP questionnaire for the Case-mix system was a guided self-administered questionnaire and consisted of 32 knowledge, attitude and practice items. The items were designed by experts in the field from the ITCC in the UKMMC. The content validity of the questionnaire was examined by four experts after item generation, and the revised questionnaire was further pre-tested. The pilot study recruited 20 health care providers at Ankara Numune Training and Research Hospital for appropriateness of language, sensitivity of questions, and average duration of administration.

The content validity was followed by factor analysis. To test the sampling size for appropriateness in factorization, the KMO test (0.92), and the Barlett test of sphericity (p<0.001) were applied. The results showed that our variables were related and suitable for factor analysis.[30] Principal component analysis and the Varimax Vertical Rotation method were used for the factor analysis. Factor loading plays a critical role in item deletion since it shows the correlation between items and their respective factors. The analyses concluded with three factors with eigenvalues >1. All factor-loading values were >0.639, and most were >0.8. Therefore, all 32 items with factor-loading values between 0.639 and 0.974 were included in the scale.

The KAP questionnaire regarding Case-mix in Turkey was found to have a strong factor structure as their factor loadings were much higher than acceptable values and the explanation rate of the three factors was adequate (76.82%). The first factor included 12 items related to attitude toward the Case-mix system that explained 36.43% of the total variance. The second factor included 10 items related to the practice of Case-mix that explained 23.39% of the total variance. The last factor comprises 10 items related to knowledge of the Case-mix system that explained 17.0% of the total variance. Factor analysis confirmed that there are no limited numbers of questions that can explain most of variance of the questionnaire.[31,32] Three factors explained 76.82% of variance; this shows that we correctly categorized the different parts of KAP into subtitles with the least overlap, factors were related to the same parts of KAP, and all suggested questions were kept in the questionnaire.[32]

In fact, the strength of any study is directly proportional with the increase of the instrument's reliability and thus its ability to detect the real significant correlations and differences in the study.[24,33] In this study the questionnaire's reliability was assessed using two tests. First, Cronbach's alpha coefficient for homogeneity was 0.941, indicating a high degree of internal consistency.[25,34] The Cronbach's alpha reliability coefficient was also calculated for the first sub-scale (knowledge) as 0.963, for the second sub-scale (attitude) as 0.964 and for the third sub-scale (practice) as 0.973. It was indicated that "the fine discriminations in the levels of the construct would be more richly reflected by an instrument with a coefficient of slightly higher than 0.8 to 0.9".[35] Second, test–retest reliability was also examined for consistency of repeated measures. Although only 60 health care providers completed the KAP test-retest, this small sample size was adequate, as the expected ICC was 0.972 (p<0.001), and there was no difference between the means of the two measurements (p>0.05). McKinley, *et al* (1997) reported that the variable and measurement technique should be the same in case of measuring test–retest reliability.[36] The time interval and the difference in the method of application may reflect on the retest scores to be lower or higher than the test scores. Nevertheless, these data confirm the adequacy of internal consistencies of the scales and high test-retest reliability of the KAP questionnaire regarding the Case-mix system in Turkey.

To our knowledge, the KAP questionnaire is the first tool developed to assess the knowledge, attitude and practice of health care providers in Case-mix and DRG systems in Turkey, and this is the first study to assess the validity and reliability of this questionnaire. This study demonstrated that the three scales of the KAP questionnaire in Case-mix and DRG systems in Turkey were highly reliable and valid. Therefore, the KAP questionnaire has acceptable indices of a standard questionnaire, and such a questionnaire can be used as an evaluation tool to assess similar health care providers in Turkey.

A few limitations exist. Although the sample size was adequate, the respondents were selected from only three hospitals in Turkey. Thus, the study result may not be easily generalized. Moreover, further validation such as criterion validity and testing on various other health care provider groups can be done to gain acceptance for this questionnaire.

## CONCLUSION

The KAP questionnaire developed to assess the knowledge, attitude and practice of health care providers in Case-mix and DRG systems in Turkey was found to have high validity and reliability. The results of this questionnaire may help to determine the levels of knowledge, attitude, practice and potential inclination of health care providers using Case-mix systems in Turkey, and may prompt a change among health care providers. Detecting the low-scoring sub-scales can contribute to developing effective early intervention policies for targeted groups. Besides, this questionnaire can provide reference data for forthcoming research regarding health care providers using these systems in Turkey.

## DECLARATION OF COMPETING INTERESTS

The authors declare they have no competing interests.

### Acknowledgments

We would like to acknowledge the expert team from the International Casemix and Clinical Coding centre for their efficient work in preparing the study tool. We are grateful to all the respondents for their time and openness during the data collection and all the team participated in collecting the data, especially Dr. Omer Kocak and Mr. Yusuf Yildiz. Special thanks for Ministry of Health, DRG Branch, Prof. Dr. İrfan Şencan, Dr. Hasan Guler and Dr. Ismail Serdaroglu. We also acknowledge Prof. Dr. Nurullah Zengin, Dr. Rabia Kahveci, Dr. Tanju Tutuncu, Mr. Ersin Gulcu and all the chief medical officers for their unlimited support.

## Appendix 1. KAP Measures and Corresponding Scales

### A. Knowledge:

1. The Case-mix system can be used to compare the efficiency of acute care hospitals.
2. Different cost weights are required for different countries in the Case-mix system.
3. A Case-mix grouper need to be customized for local use.
4. A patient's health insurance coverage affects the DRG to which the patient is assigned.
5. A digital coding tool can reduce coding errors for diagnosis and procedures.
6. Clinical coherence is one of the criteria used in the development of DRGs.
7. Case-mix grouper is a computer program which assigns diseases and procedure codes.
8. Resource usage is one of the criteria used in the development of DRGs.
9. Birth weight is required in assigning the DRG grouping.
10. Cases with expensive medications require a special Case-mix tariff.

### B. Attitude:

1. Because of the DRG system, health care providers can help patients more efficiently than before.
2. As a result of the DRG system implementation, I have to do more administrative work.
3. Using the DRG system as a health financing system would reflect positively on my income.
4. The DRG system would erode my duty of professional confidentiality.
5. The DRG system will pressure clinical staff to be more aware of the costs of treating patients.
6. The Case-mix system helps to expedite claim processing in my hospital.
7. I think that coding for diagnosis and procedures is confusing.
8. Clinicians need to put more effort into achieving the clinical pathway.
9. My hospital has adequate IT infrastructure for Case-mix and DRG system implementation.
10. My hospital has good costing data for Case-mix and DRG system implementation.
11. In my opinion, the Case-mix and DRG system did not receive the necessary support from hospital administration.
12. More efforts must be made to train staff in Case-mix and DRG system use.

### C. Practice:

1. An episode of health care is a period of inpatient care.
2. Clinical pathway is only for chronic diseases.
3. ICD-9-CM codes are used for procedure coding.
4. Patients' classification system and costing approach are the two main components of Case-mix system.
5. Coding cannot be done without patient administration data and clinical data.
6. ICD-10-CM codes are used for diagnosis coding.
7. Costing strategy in the Case-mix system starts with top down costing.
8. Physician has to provide diagnosis and procedure codes in the discharge summary.
9. A patient can have more than one DRG.
10. Cost weights are developed by Case-mix costing group.
